# Draft Genome Sequence of *Lactobacillus casei* Lbs2

**DOI:** 10.1128/genomeA.01326-14

**Published:** 2014-12-24

**Authors:** Swati Bhowmick, Mathu Malar, Abhishek Das, Bhupesh Kumar Thakur, Piu Saha, Santasabuj Das, H. M. Rashmi, Virender K. Batish, Sunita Grover, Sucheta Tripathy

**Affiliations:** a National Institute of Cholera & Enteric Diseases (NICED), West Bengal, India; b Molecular Biology Unit, Dairy Microbiology Division, National Dairy Research Institute (NDRI), Haryana, India; c Council of Scientific and Industrial Research (CSIR), Indian Institute of Chemical Biology (IICB), West Bengal, India

## Abstract

We report here a 3.2-Mb draft assembled genome of *Lactobacillus casei* Lbs2. The bacterium shows probiotic and immunomodulatory activities. The genome assembly and annotation will help to identify molecules and pathways responsible for interaction between the host immune system and the microbe.

## GENOME ANNOUNCEMENT

An imbalance between gut microbiota and the immune system is responsible for many diseases, such as inflammatory bowel diseases, metabolic disorders, and cancer (1–3). Commensal bacteria with beneficial potential have been widely marketed as probiotics (*Lactobacillus* and *Bifidobacterium* species) that promote health (4). However, several safety concerns have been raised (5) due to limited knowledge regarding the probiotic mode of action (4, 6). Identification of a potential immunomodulatory probiotic strain still remains a challenge (7–9).

*Lactobacillus casei* constitutes one of the most popularly used probiotic strains marketed throughout the world, e.g., *L. casei* Shiorta (Yakult Honsha, Japan) and *L. casei* Immunitas (Actimel). Although complete *L. casei* genome sequences (NC_008526.1, NC_014334.1, NC_010999.1, NC_017474.1, NC_017473.1, NC_018641.1, and NC_021721) are available and genome analysis has elucidated its symbiotic mechanisms (10), information is still lacking on the immunomodulatory molecules of *L. casei*. We are reporting genome sequences of a novel strain, Lbs2, which is an indigenous isolate of healthy Indian gut identified by 16S rRNA sequencing (KM203837) and available at the probiotic repository established at the Molecular Biology Unit, NDRI, Karnal (India). The strain possesses probiotic attributes, such as acid and bile tolerance, hydrophobicity, and auto- and coaggregation properties. Intragastric administration of Lbs2 to mice induced a strong regulatory response with the generation of CD103^+^ dendritic cells (DCs) and FoxP3^+^ T-regulatory (Treg) cells in the mesenteric lymph nodes and intestinal lamina propria. High titers of regulatory cytokines (interleukin 10 [IL-10] and transforming growth factor-β [TGF-β]) were observed in the serum and colonic tissue lysates. As extracellular proteins of bacteria interact directly with mucosal cells (11), we analyzed proteins secreted in culture supernatant of Lbs2 through SDS-PAGE coupled with a matrix-assisted laser desorption ionization–time of flight tandem mass spectrometry (MALDI-TOF/TOF) mass spectrometer. A MASCOT search revealed high-confidence identification of four proteins, extracellular transglycosylase (YP_004888342), adherence protein (YP_004889513), and two moonlighting proteins, glyceraldehyde-3-phosphate dehydrogenase (YP_003062238.1) and lactate dehydrogenase (1LLC). Genome analysis will be used to determine molecular mechanisms in immunomodulation of this potent strain.

Whole-genome sequencing of *L. casei* Lbs2 was carried out with 200 µL DNA with a concentration of 219.4 ng/µl. A paired-end library was constructed with an insert size of approximately 300 bp, generating 151-bp reads at 500 coverage. Additionally, Illumina mate-pair libraries with a 3-kb insert size and 101-bp reads generated 150× coverage. Sequences were quality processed using the fastQC pipeline and in-house scripts. Approximately 9.6 million reads from paired-end and 4.4 million reads from mate-pair libraries were assembled initially using IDBA (12). IDBA assembly was randomly sheared, generating 6 k mate-pair jump data at 10× and 101-bp read length. Allpaths-LG-49856 (13) was used, combining paired-end data and mate-pair (3 kb and 6 kb) data producing the final assembly. The final assembly is 3.2 Mb with 297 scaffolds; the genome *N*_50_ is 48,309; and the largest scaffold is 185,255 bp and the smallest is 1,682 bp. The sequences were annotated at IICB and the Prokaryotic Genomes Annotation Pipeline (PGAAP) application on the NCBI server (http://www.ncbi.nlm.nih.gov/genomes/static/Pipeline.html), and both annotations concurred. The final annotated assembly has about 2,894 structural genes, 2,453 coding sequences (CDSs), 402 pseudogenes, 8 rRNAs, 29 tRNAs, and 2 ncRNAs.

This genome sequence and annotation hold great promise for the discovery of novel probiotic effector molecules.

### Nucleotide sequence accession number.

The genome sequences are now available at NCBI under the accession number JPKN00000000.
